# Inclusion of mobile telephone numbers into an ongoing population health survey in New South Wales, Australia, using an overlapping dual-frame design: impact on the time series

**DOI:** 10.1186/1756-0500-7-517

**Published:** 2014-08-12

**Authors:** Margo L Barr, Raymond A Ferguson, David G Steel

**Affiliations:** Centre for Epidemiology and Evidence, NSW Ministry of Health, 73 Miller Street, North Sydney, Australia; National Institute for Applied Statistics Research Australia, University of Wollongong, Wollongong, Australia

**Keywords:** Telephone health survey, Sample survey, Overlapping dual-frame, Time series

## Abstract

**Background:**

Since 1997, the NSW Population Health Survey (NSWPHS) had selected the sample using random digit dialing of landline telephone numbers. When the survey began coverage of the population by landline phone frames was high (96%). As landline coverage in Australia has declined and continues to do so, in 2012, a sample of mobile telephone numbers was added to the survey using an overlapping dual-frame design. Details of the methodology are published elsewhere. This paper discusses the impacts of the sampling frame change on the time series, and provides possible approaches to handling these impacts.

**Methods:**

Prevalence estimates were calculated for type of phone-use, and a range of health indicators. Prevalence ratios (PR) for each of the health indicators were also calculated using Poisson regression analysis with robust variance estimation by type of phone-use. Health estimates for 2012 were compared to 2011. The full time series was examined for selected health indicators.

**Results:**

It was estimated from the 2012 NSWPHS that 20.0% of the NSW population were mobile-only phone users. Looking at the full time series for *overweight or obese* and *current smoking* if the NSWPHS had continued to be undertaken only using a landline frame*, overweight or obese* would have been shown to continue to increase and *current smoking* would have been shown to continue to decrease. However, with the introduction of the overlapping dual-frame design in 2012, *overweight or obese* increased until 2011 and then decreased in 2012, and *current smoking* decreased until 2011, and then increased in 2012. Our examination of these time series showed that the changes were a consequence of the sampling frame change and were not real changes. Both the backcasting method and the minimal coverage method could adequately adjust for the design change and allow for the continuation of the time series.

**Conclusions:**

The inclusion of the mobile telephone numbers, through an overlapping dual-frame design, did impact on the time series for some of the health indicators collected through the NSWPHS, but only in that it corrected the estimates that were being calculated from a sample frame that was progressively covering less of the population.

## Background

Since 2002, information about the health of the NSW population***—***7.3 million***—***has been obtained using the NSW Population Health Survey (NSWPHS) [1]. This survey is a continuous sample survey of approximately 15,000 persons each year; with equal numbers selected from each of the strata ***—***health administrative areas***—***using random digit dialing (RDD) of landline telephone numbers and computer assisted telephone interviewing (CATI). Prior to 2002, two surveys were conducted in 1997 and 1998. When the survey began in 1997 coverage of the Australian population by landline phone frames was estimated to be 96%, however by 2006 coverage had dropped to 91% and by 2009 coverage had declined even further to 85% [2, 3]. This decline in the coverage of the population by landline phone frames was consistent with what was occurring in other countries [4–6].

Although not routinely available in Australia, differences in health risk factor and health status prevalence estimates between adults covered in a landline phone frame, and adults who live in a household without a landline telephone connection on which to make and answer calls (ie mobile-only phone users), have been measured in the USA using the National Health Interview Survey (NHIS) since 2003 [7]. This survey found substantial differences for the health indicators: *five or more drinks in one day at least once in the past year* (17.5% v 30.5% - 74% higher), *current smokers* (14.5% v 24.3% - 68% higher), and *ever diagnosed with diabetes* (10.8% v 6.2% - 43% lower) [8].

Based on this overseas experience, it was estimated that when the overall landline phone coverage dropped below 85% in Australia, the differences in health indicators between people who were covered in the landline phone frame and mobile-only phone users differed by more than 50%, and/or there were associations between phone usage and the health indicators, it would impact appreciably on the overall NSW prevalence estimates [9]. In 2010, the landline phone coverage in Australia dropped below 85%, and an Australian study showed relative differences between those people who were covered by the landline phone frame, and mobile-only phone users, of 69% for *current smoking* (20.4% v 34.5%) and 56% *for ever diagnosed with diabetes* (11.7% v 5.2%) [10]. This study also showed that for *current smoking*, even after adjusting by age and sex, the mobile-only phone users were more likely to be *current smokers*. So, because of these changes in landline phone coverage, mobile telephone numbers were included in the 2012 NSWPHS, using an overlapping dual-frame design [11]. Within a stratum the landline sample was selected using equal probability of selection of landline telephone numbers and then random selection of one person from the selected household. In the mobile phone sample an equal probability sample of mobile telephone numbers in Australia was selected and screened for adult residents in NSW. If the respondent had one or more children one child was selected at random. Sample weights thus reflected the differing sampling probabilities. The sample weights of the dual phone-users were then adjusted so that the composite factor used to combine the estimates for this component obtained from the landline sample and the mobile phone sample, λ, was set at 0.5. Benchmarking to the reference population was then performed by adjusting the weights for differences between weighted estimates of the age and sex structure obtained from the combined landline and mobile phone sample and ABS mid-year population estimates for each stratum. More details about the weighting strategy are provided in Barr et al. [12].

This paper firstly provides prevalence estimates for type of phone-use, health risk factor and health status from the 2012 NSWPHS. It also examines if, as other authors have found, there are any associations between the health indicators and type of phone-use, adjusting for the weighting variables. Health estimates from the 2012 NSWPHS, and the landline frame sample, re-benchmarked to the NSW population, were then compared to the previous year’s estimates. The impacts on the time series of the change in design to an overlapping dual-frame design is then discussed as are possible approaches to handling these impacts.

## Methods

### Data source

Data from the NSWPHS for 2012 was used. This consisted of data on 15,214 respondents with 10,518 (69.1%) from the landline phone frame, of which 1,792, (17.0%) were landline only, and 4,696 (30.1%) from the mobile phone frame, of which 1,121 (23.9%) were mobile only. The overall response rate was 31.0%, co-operation rate was 63.4%, refusal rate was 17.9% and contact rate was 66.9%.

### Calculation of prevalence estimates

Estimates for type of phone-use were calculated overall and by selected demographic characteristics. Health risk factor and health status indicators were selected from the questions asked in the survey as shown in Table 1. Prevalence estimates and 95 per cent confidence intervals using the SURVEYFREQ procedure in SAS, which uses the Taylor expansion method to calculate sampling errors of complex sample designs, were calculated for each indicator [13].

**Table 1:** **Health indicators definitions and questions, 2102 NSWPHS**

Health indicator	Definition	Question/s
Five or more drinks of alcohol in a day	The indicator includes those who drink five or more standard drinks on a day when they drink alcohol.	How often do you usually drink alcohol?
		On a day when you drink alcohol, how many standard drinks do you usually have?
		A standard drink is equal to 1 middy of full-strength beer, 1 schooner of light beer, 1 small glass of wine, or 1 pub-sized nip of spirits.
More than two alcoholic drinks in a day	The indicator includes those who drink more than two standard drinks on a day when they drink alcohol.	How often do you usually drink alcohol?
		On a day when you drink alcohol, how many standard drinks do you usually have?
		A standard drink is equal to 1 middy of full-strength beer, 1 schooner of light beer, 1 small glass of wine, or 1 pub-sized nip of spirits.
Recommended fruit intake	The indicator includes those who consumed two or more serves of fruit a day. The recommended fruit intake is at least 2 serves a day for persons aged 19 years and over, depending on their overall diet. For simplification, this recommendation is applied to 16-18 year olds. One serve is equivalent to 1 medium piece or 2 small pieces of fruit.	How many serves of fruit do you usually eat each day?
Recommended vegetable intake	The indicator includes those who consumed 5 or more serves of vegetables a day. The recommended vegetable intake is at least 5 serves a day for persons aged 16 years and over, depending on their overall diet. One serve is equivalent to 1/2 cup of cooked vegetables or 1 cup of salad vegetables.	How many serves of vegetables do you usually eat each day?
Current smoking	The indicator includes those who smoked daily or occasionally.	Which of the following best describes your smoking status: smoke daily, smoke occasionally, do not smoke now but I used to, I have tried it a few times but never smoked regularly, or I have never smoked?
Adequate physical activity	The indicator includes those who did adequate physical activity. Adequate physical activity is a total of 150 minutes a week on 5 separate occasions. The total minutes were calculated by adding minutes in the last week spent walking continuously for at least 10 minutes, minutes doing moderate physical activity, plus 2 x minutes doing vigorous physical activity.	In the last week, how many times have you walked continuously for at least 10 minutes for recreation or exercise or to get to or from places?
		What do you estimate was the total time you spent walking in this way in the last week?
		In the last week, how many times did you do any vigorous physical activity that made you breathe harder or puff and pant?
		What do you estimate was the total time you spent doing this vigorous physical activity in the last week?
		In the last week, how many times did you do any other more moderate physical activity that you have not already mentioned?
Positive self-reported health status	The indicator includes those responding excellent, very good, or good to a global self-rated health status question.	Overall, how would you rate your health during the last 4 weeks: Was it excellent, very good, good, fair, poor, or very poor?
Current asthma	The indicator includes those who had symptoms of asthma or treatment for asthma in the last 12 months.	Have you ever been told by a doctor or hospital you have asthma?
		Have you had symptoms of asthma or treatment for asthma in the last 12 months?
Ever diagnosed with diabetes	The indicator includes those who either had diabetes or high blood glucose but did not have gestational diabetes.	Have you ever been told by a doctor or hospital you have diabetes?
		Have you ever been told by a doctor or hospital you have high blood glucose?
		If female, Were you pregnant when you were first told you had diabetes or high blood glucose?
		Have you ever had diabetes or high blood glucose apart from when you were pregnant?
Overweight or obese	The indicator includes those who are overweight or obese: that is with a Body Mass Index (BMI) of 25.0 or higher. BMI is calculated as follows: BMI = weight (kg)/height (m)^2^. Categories for this indicator include overweight (BMI from 25.0 to 29.9) and obese (BMI of 30.0 and over).	How tall are you without shoes?
		How much do you weigh without clothes or shoes?

### Associations between the health indicators and type of phone-use

Prevalence ratios (PR) for each of the health indicators were calculated using Poisson regression analysis with robust variance estimation by type of phone-use using the categories mobile-only, landline-only, dual phone users in the mobile frame, and dual phone users in the landline frame as the reference category. This analysis was then repeated adjusting for all of the weighting variables including age group, sex, administration area, number of eligible persons in the household, and number of phone lines.

This analysis used the GENMOD procedure in SAS. As the Poisson model uses the natural logarithm as the link function, exponentiation of the parameter estimates was used to obtain the PRs for the study factors [14–16].

### Comparison of 2012 prevalence estimates with previous years

Estimates for health related variables for the 2012 NSWPHS, as well as using just the landline frame sample, re-benchmarked to the NSW population, were then compared to the 2011 NSWPHS. Significant differences were identified by comparing the differences between the two estimates, divided by the standard error of the differences, calculated as √[SE(E_2011PHS_)^2^ + SE(E_2012PHS_)^2^]), with the standard normal distribution [17]. The full time series was examined for health indicators where there was an association between type of phone-use and the indicator, and both significance and direction changed between sampling designs.

Two solutions were considered to adjust the time series for the expansion of the coverage of the survey. The first being the backcasting method [18] where the 2012 figures were used and a correction factor was applied to each of the proceeding annual time points using the formula  where  is the estimate from the landline phone frame,  is the revised estimate, *A* is the landline frame, and *b* is the mobile-only phone users. The difference  is the relative difference measured in this study, which is _,_ and  is the coverage each year as reported by the Australian Communication and Media Authority (ACMA) [2, 3]. The second method was the minimal coverage method, which only allowed inclusion of point estimates into the time series where there was adequate population coverage, with adequate population coverage being defined as 85% or above [9].

## Results

### Prevalence estimates for 2012

It was estimated from the 2012 NSWPHS that 20.0% (95%CI 18.3%-22.0%) of the NSW population were mobile-only phone users, 9.6% (95%CI 8.8%-10.4%) were landline only, and 70.2% (95%CI 68.4%-72.1%) were dual phone users. Table 2 shows estimates for type of phone-use overall, and, for selected demographic characteristics. As shown in Table 2, mobile-only phone user rates were highest in young people, unmarried people, and Aboriginal and/or Torres Strait Islander peoples.

**Table 2:** **Type of phone-use estimations for NSW from 2012 NSWPHS**

Demographic groups		Landline-only		Mobile-only		Dual phone users		Landline phone users – who may also have a mobile phone	
		%	95%CI	%	95%CI	%	95%CI	%	95%CI
Sex	Males	9.6	8.4-10.8	20.9	18.4-23.4	69.5	66.9-72.1	79.1	76.6-81.6
	Females	9.5	8.4-10.7	19.5	16.9-22.1	71.0	68.4-73.6	80.5	77.9-83.2
Age group	14-24 years	1.6	0.9-2.4	21.1	17.5-24.7	77.3	73.6-81.0	78.9	75.3-82.5
	25-34 years	0.7	0.3-1.1	45.1	39.4-50.8	54.2	48.5-59.9	54.9	49.2-60.6
	35-44 years	4.2	2.8-5.5	24.2	19.1-29.2	71.6	66.7-76.6	75.8	70.8-80.9
	45-54 years	7.2	5.2-9.2	14.3	11.0-17.7	78.5	74.7-82.2	85.7	82.3-89.0
	55-64 years	10.6	8.7-12.5	11.6	7.2-16.0	77.7	73.4-82.1	88.3	84.0-92.8
	65 + years	33.6	30.6-36.7	3.2	2.0-4.3	63.1	60.1-66.2	96.7	95.7-98.0
Aboriginal and/or Torres Strait Islanders		7.6	4.2-11.1	38.7	29.9-47.5	53.7	45.2-62.2	61.3	52.5-70.1
Never married		3.4	2.7-4.1	33.4	29.8-37.0	63.2	59.6-66.8	66.6	63.0-70.2
Separated but not divorced		9.8	5.0-14.7	31.7	19.7-43.8	58.4	47.2-69.6	68.2	56.2-80.3
Born overseas		7.8	6.6-9.0	23.2	20.0-26.3	69.1	65.9-72.2	76.9	73.7-80.0
Low household income (<$20,000)		21.0	18.2-23.6	26.6	21.9-31.4	52.4	48.3-56.6	73.4	68.6-78.1
**NSW OVERALL**		**9.6**	**8.8-10.4**	**20.2**	**18.3-22.0**	**70.2**	**68.4-72.1**	**79.8**	**78.0-81.7**

With regard to health risk factor and health status indicators, it was estimated from the dual frame 2012 NSWPHS as shown in Table 3, that 11.1% (95%CI 9.9%-12.2%) of the population *drank five or more drinks of alcohol in a day*, 27.6% (95%CI 25.9%-29.3%) *drank more than two alcoholic drinks in a day*, 53.4% (95%CI 51.5%-55.3%) *met the recommended fruit intake*, 10.0% (95%CI 8.8%-11.1%) *met the recommended vegetable intake*, 17.1% (95%CI 15.6%-18.6%) were *current smokers*, 56.2% (95%CI 54.2%-58.1%) *did adequate physical activity*, 82.4% (95%CI 81.2%-83.6%) *had positive self-rated health status*, 10.1% (95%CI 9.1%-11.1%) *had current asthma*, 8.4% (95%CI 7.5%-9.2%) *were ever diagnosed with diabetes*, and 49.7% (95%CI 47.7%-51.6%) *were overweight or obese*.

**Table 3:** **Health indicators estimate comparisons between adults with landline phones, who may also have a mobile phone, and mobile-only phone users, 2012 NSWPHS**

Health indicators	Adults with landline phones - who may also have a mobile phone	Mobile-only phone users	Relative difference % (95%CI)	Total % (95%CI)	
Five or more drinks of alcohol in a day	9.0%	19.3%	114% (93%, 130%)	11.1%	(9.9%-12.2%)
More than two alcoholic drinks in a day	25.6%	35.0%	37% (28%, 44%)	27.6%	(25.9%-29.3%)
Recommended fruit intake	53.7%	52.0%	-3% (-10%, 3%)	53.4%	(51.5%-55.3%)
Recommended vegetable intake	10.5%	7.8%	-26% (-85%, -1%)	10.0%	(8.8%-11.1%*)*
Current smoking	14.0%	28.3%	103% (86%, 117%)	17.1%	(15.6%-18.6%)
Adequate physical activity	53.2%	66.7%	26% (21%, 29%)	56. 2%	(54.2%-58.1%)
Positive self-reported health status	81.4%	86.0%	6% (4%, 7%)	82.4%	(81.2%-83.6%*)*
Current asthma	10.7%	8.1%	-25% (-17%, -34%)	10.1%	(9.1%-11.1%*)*
Ever diagnosed with diabetes	9.3%	5.2%	-44% (-62%, -30%)	8.4%	(7.5%-9.2%)
Overweight or obese	52.1%	41.0%	-21% (-29%, -14%)	49.7%	(47.7%-51.6%)

### Associations between the health indicators and type of phone-use

Table 3 also shows the health indicator prevalence estimates for the 2012 NSWPHS overall, and for those with landline phones, who may also have a mobile phone, and those who are mobile-only phone users. As shown in Table 3 there were relative differences of more than 50% for *five or more drinks of alcohol in a day* (9.0% v 19.3%, 114% higher), and *current smoking* (14.0% v 28.3%, 103% higher).

Table 4 shows null PRs and adjusted PRs, for weighting variables, by type of phone-use for each of the selected health indicators from the 2012 NSWPHS. As shown in Table 4, after adjusting by the weighting variables of age group, sex, administration area, number of phone lines, and number of eligible persons in the household, mobile-only phone users were more likely to: *drink five or more drinks of alcohol in a day* (PR, 1.29; 95%CI, 1.04-1.59) and *be current smokers* (PR, 1.39; 95%CI, 1.20-1.63), and mobile-only phone users were less likely to *meet the recommended vegetable intake* (PR, 0.65; 95%CI 0.50-0.85) and *be overweight or obese* (PR, 0.90; 95%CI 0.83-0.97) than dual phone users from the landline frame. Also, after adjusting by the weighting variables of age group, sex, administration area, number of phone lines, and number of eligible persons in the household, dual phone users from the mobile frame were significantly different to the dual phone users from the landline frame: for *more than two drinks of alcohol in a day* (PR 0.88; 95%CI 0.80-0.97), *current smoking* (PR, 0.85; 95%CI 0.74-0.98), and *current asthma* (PR 0.77; 95%CI 0.65-0.91) which further supports the use of overlapping dual-frame designs, rather than screening dual-frame designs.

**Table 4:** **Null and adjusted Prevalence Ratios (PR) for selected indicators using Poisson regression analysis with robust variance estimation, 2012 NSWPHS**

Indicator	Category	Null				Adjusted by weighting variables^			
		PR 95%CI				PR 95%CI			
Five or more drinks of alcohol in a day	Dual phone users (mobile frame)	1.48	1.29	1.69	*	0.94	0.78	1.13	
	Landline-only phone user	0.86	0.70	1.06		1.25	1.02	1.54	*
	Mobile-only phone user	2.51	2.14	2.93	*	1.29	1.04	1.59	*
	REF – Dual phone users (landline frame)	1.00				1.00			
More than two drinks of alcohol in a day	Dual phone users (mobile frame)	1.18	1.10	1.27	*	0.88	0.80	0.97	#
	Landline-only phone user	0.71	0.63	0.80	*#*	0.89	0.79	0.99	*#*
	Mobile-only phone user	1.63	1.49	1.79	*	1.07	0.95	1.20	
	REF – Dual phone users (landline frame)	1.00				1.00			
Recommended fruit intake	Dual phone users (mobile frame)	0.94	0.90	0.97	#	0.99	0.94	1.04	
	Landline-only phone user	0.99	0.95	1.04		0.96	0.92	1.01	
	Mobile-only phone user	0.87	0.81	0.92	#	0.93	0.86	1.01	
	REF – Dual phone users (landline frame)	1.00				1.00			
Recommended vegetable intake	Dual phone users (mobile frame)	0.68	0.60	0.77	#	0.87	0.74	1.02	
	Landline-only phone user	0.86	0.74	0.99	#	0.82	0.71	0.96	#
	Mobile-only phone user	0.47	0.37	0.60	#	0.65	0.50	0.85	#
	REF – Dual phone users (landline frame)	1.00				1.00			
Current smoking	Dual phone users (mobile frame)	1.19	1.08	1.32	*	0.85	0.74	0.98	#
	Landline-only phone user	1.03	0.91	1.18		1.34	1.18	1.53	*
	Mobile-only phone user	2.10	1.88	2.35	*	1.39	1.20	1.63	*
	REF – Dual phone users (landline frame)	1.00				1.00			
Adequate physical activity	Dual phone users (mobile frame)	1.10	1.05	1.14	*	0.96	0.91	1.02	
	Landline-only phone user	0.77	0.72	0.82	#	0.84	0.78	0.89	#
	Mobile-only phone user	1.26	1.19	1.32	*	1.04	0.97	1.12	
	REF – Dual phone users (landline frame)	1.00				1.00			
Positive self-reported health status	Dual phone users (mobile frame)	1.05	1.03	1.07	*	1.04	1.00	1.07	
	Landline-only phone user	0.86	0.84	0.89	#	0.89	0.86	0.92	#
	Mobile-only phone user	1.05	1.02	1.08	*	1.01	0.97	1.05	
	REF – Dual phone users (landline frame)	1.00				1.00			
Current asthma	Dual phone users (mobile frame)	0.72	0.63	0.82	#	0.77	0.65	0.91	#
	Landline-only phone user	0.98	0.86	1.13		1.02	0.88	1.17	
	Mobile-only phone user	0.78	0.64	0.94	#	0.82	0.66	1.04	
	REF – Dual phone users (landline frame)	1.00				1.00			
Ever diagnosed with diabetes	Dual phone users (mobile frame)	0.69	0.60	0.80	#	1.01	0.86	1.20	
	Landline-only phone user	1.63	1.45	1.83	*	1.23	1.09	1.38	*
	Mobile-only phone user	0.51	0.40	0.66	#	1.13	0.86	1.49	
	REF – Dual phone users (landline frame)	1.00				1.00			
Overweight or obese	Dual phone users (mobile frame)	0.88	0.84	0.91	#	0.99	0.94	1.04	
	Landline-only phone user	1.02	0.98	1.07		0.97	0.93	1.02	
	Mobile-only phone user	0.73	0.68	0.78	#	0.90	0.83	0.97	#
	REF – Dual phone users (landline frame)	1.00				1.00			

NOTE: *significantly (p < 0.05) higher than reference category; #significantly (p < 0.05) lower than reference category.

NOTE: ^weighting variables; age group, sex, health administration area, household size and number of telephone lines.

### Comparison of 2012 prevalence estimates with previous years

Table 5 shows the health indicators estimates from the 2012 NSWPHS, compared to the 2011 NSWPHS. Significantly higher estimates were found in 2012 for: *recommended fruit intake* (from 50.4% to 53.4%, p = 0.016), *recommended vegetable intake* (from 8.4% to 10.0%, p = 0.026), *current smoking* (from 14.7% to 17.1%, p = 0.011), *positive self-reported health status* (from 80.3% to 82.4%, p = 0.010), and significantly lower estimates for *overweight or obese* (52.2% to 49.7%, p = 0.047).

**Table 5:** **Health indicators estimate comparisons by year, NSWPHS**

Health indicators	2011 NSWPHS		2012 NSWPHS Dual-frame		2012 NSWPHS Landline frame		Prevalence difference					
							2012 NSWPHS (dual frame) minus 2011 NSWPHS			2012 NSWPHS (landline frame) minus 2011 NSWPHS		
	%	SE %	%	SE %	%	SE %	Prev diff % (95%CI)	p-value		Prev diff % (95%CI)	p-value	
Five or more drinks of alcohol in a day	11.3	1.10	11.1	0.59	9.4	0.81	-0.2 (0.4,-0.8)	0.432		-1.9 (-1.6, -2.2)	0.083	
More than two alcoholic drinks in a day	29.6	0.74	27.6	0.87	27.1	1.23	-2.0 (-2.14, -1.9)	0.092		-2.5 (-3.0, -2.0)	0.042	#
Recommended fruit intake	50.4	0.74	53.4	0.98	55.9	1.27	3.0 (2.75, 3.3)	0.016	*	5.5 (5.0, 6.0)	<0.001	*
Recommended vegetable intake	8.4	0.35	10.0	0.60	12.3	0.94	1.6 (1.2, 2.0)	0.026	*	3.9 (3.2, 4.6)	<0.001	*
Current smoking	14.7	0.55	17.1	0.75	14.4	0.92	2.4 (2.2, 2.6)	0.011	*	-0.3 (-0.7, 0.1)	0.373	
Adequate physical activity	54.6	0.75	56.2	0.99	56.8	1.30	1.6 (1.4, 1.9)	0.224		2.2 (1.7, 2.7)	0.069	
Positive self-reported health status	80.3	0.56	82.4	0.60	80.6	0.95	2.1 (2.1, 2.2)	0.010	*	0.3 (-0.1, 0.7)	0.381	
Current asthma	11.3	0.46	10.1	0.52	12.6	0.97	-1.2 (-1.3, -1.1)	0.079		1.3 (0.7, 1.9)	0.122	
Ever diagnosed with diabetes	8.1	0.31	8.4	0.44	8.6	0.54	0.3 (0.1, 0.5)	0.573		0.5 (0.2, 0.9)	0.215	
Overweight or obese	52.2	0.76	49.7	1.00	53.9	1.35	-2.5 (-2.8,-2.3)	0.047	#	1.7 (1.1, 2.3)	0.138	

NOTE *significantly (p < 0.05) higher than comparison group; #significantly (p < 0.05) lower than comparison group.

Table 5 also shows the health indicators estimates, using just the landline frame sample for 2012, re-benchmarked to the NSW population, compared to the 2011 NSWPHS. Significantly higher estimates were again found *for recommended fruit intake* (from 50.4% to 55.9%, p < 0.001) and *recommended vegetable intake* (from 8.4% to 12.3%, p < 0.001), and significantly lower estimates for *more than two alcoholic drinks in a day* (29.6% to 27.1%, p = 0.042). However *current smoking, positive self-reported health status*, and *overweight or obese* were no longer statistically significantly different, and the difference had changed in direction for *current smoking* and *overweight or obese*.

Table 6 shows a summary of the factors used to predict if the sampling frame change is likely to impact on the time series. These factors are; 50% difference or more for non-landline frame persons, association between type of phone-use and the indicator, change in significance between sampling designs, and change in direction between sampling designs. Based on this analysis, the two indicators for which the time series was most likely to be affected, were *current smoking* and *overweight or obese*.

**Table 6:** **Summary of the factors used to predict if the design change was likely to impact on the time series**

Health indicators	50% or more different for non-landline frame	Association between phone usage and indicator	Sig diff 2011 and 2012 (dual- frame)	Sig diff 2011 and 2012 (landline frame)	Change in significance between sampling designs	Change in direction between sampling designs
Five or more drinks of alcohol in a day	√	√				
More than two alcoholic drinks in a day				√	√	
Recommended fruit intake			√	√		
Recommended vegetable intake		√	√	√		
Current smoking	√	√	√		√	√
Adequate physical activity						
Positive self-reported health status			√		√	
Current asthma						
Ever diagnosed with diabetes						
Overweight or obese		√	√		√	√

**Figure 1:**
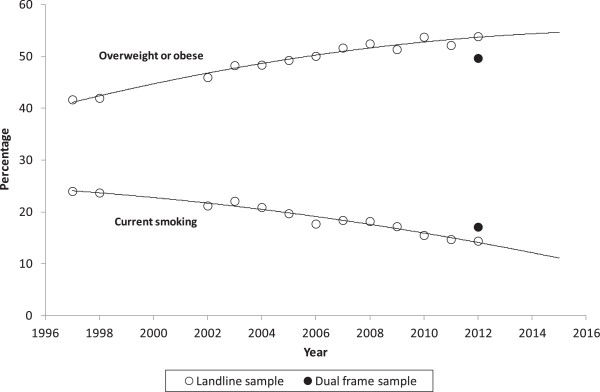
**Landline sample time series estimates for current smoking and overweight or obese from the NSWPHS compared to the estimates from the dual-frame for 2012 NSWPHS.**

Looking at the full time series as shown in Figure 1, if the NSWPHS had continued to be undertaken only using a landline frame*, overweight or obese* would have been shown to continue to increase and *current smoking* would have been shown to continue to decrease. However, with the introduction of the overlapping dual-frame design in 2012, *overweight or obese* increased until 2011 and then decreased in 2012, and *current smoking* decreased until 2011, and then increased in 2012.

### Adjusting the time series

**Figure 2:**
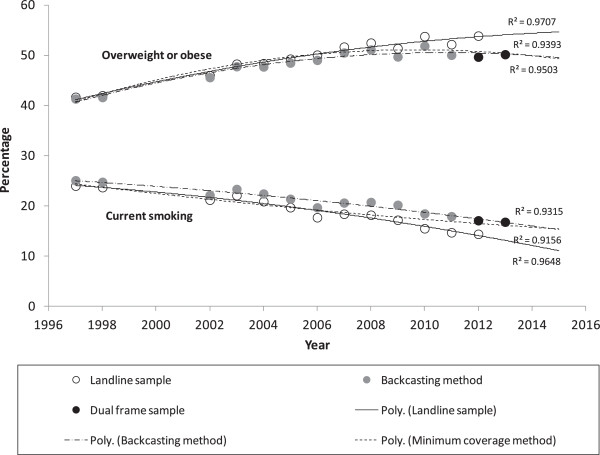
**Options for adjusting the time series estimates for current smoking and overweight or obese from the NSWPHS to incorporate the dual-frame sample from 2012 onwards.**

The backcasting method, applying relative differences of 103% for *current smoking* and -21% for *overweight or obese* across all years and landline coverage of 96% from 1997-2002, 95% in 2003, 93.5% in 2004, 92.4% in 2005, 90.1% in 2006, 89.3% in 2007, 87.6% in 2008, 84.6% in 2009, 83.1% in 2010, 80.6% in 2011 and 77.8% in 2012, resulted, as shown in Figure 2, in the trend for *current smoking* continuing to decrease in 2012 and the trend for *overweight or obese* increasing until 2008, and plateauing thereafter. Lines of best fit were: y = -0.0112x^2^ + 44.411x – 43981, R^2^ = 0.9315 for *current smoking*, and y = -0.0523x^2^ + 210.18x – 211256, R^2^ = 0.9503 *for overweight or obese* where x = year-1996.

The minimal coverage method, removing years 2010 and 2011 when the population coverage was less than 85% resulted, as shown in Figure 2, in the trend for *current smoking* continuing to decrease in 2012, but at a lesser rate and the trend for *overweight or obese* increasing until 2008 and plateauing thereafter. Lines of best fit were: y = -0.0112x^2^ - 0.3169x + 25.34, R^2^ = 0.9156 for *current smoking*, and y = -0.0641x^2^ + 1.7555x + 39.093, R^2^ = 0.9393 for *overweight or obese* where x = year-1996.

Preliminary estimates for the first quarter of 2013 were also included for *current smoking* and *overweight or obese*; to examine which of the adjustment methods would best predict the 2013 estimates. Both methods were very close for *overweight and obese*, and the backcasting method was slightly better for *current smoking*. However, both methods had similar trajectories into the future and both were getting further away from the landline frame trajectory.

## Discussion

We had previously found that the dual-frame gave a more representative sample [11]. The type of phone-use estimates from the 2012 NSWPHS were similar to those published for Australia in 2012 by ACMA from the Roy Morgan Single Source Survey***—***19.9% for mobile-only, 8.0% for landline-only and 69.8% for dual phone users [19].

In this study, we found relative differences of over 50% between people who were covered by the landline frame and mobile-only phone users for health indicators: *five or more drinks of alcohol in a day*, and *current smoking*. We also found that type of phone-use was associated with many of the health indicators, in particular mobile-only phone users were significantly different for: *drink five or more drinks of alcohol in a day, current smoking, recommended vegetable intake,* and *overweight or obese*, even after adjusting for the weighting variables. Our results were consistent with other studies [10, 20, 21].

When we compared the health indicators estimates from the 2012 NSWPHS to the 2011 NSWPHS, we found significant differences for *recommended fruit intake, recommended vegetable intake, current smoking, positive self-reported health status,* and *overweight or obese*. However, when we compared the health indicators estimates using only the landline frame sample, re-benchmarked to the NSW population, to the 2011 NSWPHS, *current smoking, positive self-reported health status,* and *overweight or obese* were no longer significantly different, and the difference had changed in direction, for *current smoking* and *overweight or obese*.

So, how do we interpret these changes? Did *current smoking* really increase in 2012, and did *overweight or obese* really decrease in 2012, or is it a consequence of the design change? Our examination of the time series for *current smoking* and *overweight or obese* showed that it was a consequence of the design change and not a real change.

The backcasting method was best able to predict the 2013 figures for *current smoking*. This method appears superior to the minimal coverage method in that it not only corrects the years when the landline frame coverage was sub-optimal, but it also adjusts the estimates to what they should have been for all the other years, if mobile-only phone users were included. However the backcasting method also requires the making of several assumptions, that being that the relative difference between people covered by the landline phone frame and mobile-only phone users has remained constant over time, and, that the landline phone coverage estimates for Australia were appropriate for NSW. Also, for the backcasting method, a more complex formula would need to be used if it was being used for demographic groups***—***requiring  for each group which is not currently available***—***and could quite quickly become very complex with numerous assumptions needing to be made. The minimal coverage method does not require any additional assumptions to be made; it just requires a decision on what is considered adequate population coverage by the sample frame or frames.

Figure 2 shows that the difference between the landline frame time series and the adjusted dual-frame time series, for *current smoking* and *overweight or obese*, are widening over time. A recent study on the European telephone surveys has concluded that coverage bias from surveys using only landline frames in Europe are increasing over time [22]. This study highlighted the need for mobile telephone number augmentation of the sample to occur prior to the landline phone coverage becoming sub-optimal.

## Conclusions

The inclusion of the mobile telephone numbers through an overlapping dual-frame design did impact on the time series for some of the health risk factors and health status estimates, in that it corrected the estimates that were being calculated from a sample frame, which was getting progressively less representative of the population. Therefore, continuing to use only landline frames in Australia, although maintaining the same design, is not keeping the estimates the same because of the decreasing coverage of landline frames.

### Key message

Health estimates from surveys using only landline frame sampling are progressively getting “further from the truth”.

## Authors’ information

MLB is a PhD student with the National Institute for Applied Statistics Research, University of Wollongong, Wollongong, Australia.

## Abbreviations

CATI: Computer assisted telephone interviewing
NHIS: National Health Interview Survey
NSW: New South Wales
NSWPHS: NSW Population Health Survey
PR: Prevalence ratio
RDD: Random digit dialing
RMSSS: Roy Morgan Single Source Survey.
